# Supplementary material for: Effect of Tricarboxylic Acids on the Formation of Hydrogels with Melem or Melamine: Morphological, Structural and Rheological Investigations
Source: Gels. 2022 Jan 10;8(1):51. doi: 10.3390/gels8010051 (PMC8774776; doi:10.3390/gels8010051)
Supplement: Supplementary file 1 [file gels-08-00051-s001.zip › gels-1508750-supplementary.pdf]

Supplementary Materials

Effect of Tricarboxylic Acids on the Formation of Hydrogels with Melem or Melamine: Morphological, Structural and Rheological Investigations

Pradip Kumar Sukul, Puspendu Das, Gopal Lal Dhakar, Lalmohan Das and Sudip Malik

[1] School of Applied and Interdisciplinary Sciences, Indian Association for the Cultivation of Science, 2A & 2B Raja S. C. Mullick Road, Jadavpur, Kolkata – 700032, India.

[2] Department of Chemistry, Amity Institute of Applied Sciences, Amity University Kolkata, Action Area-II, Kadampukur, New Town, Rajarhat, Kolkata-700135, India

1. Materials and Instruments:

4-Bromoacetophenone, $K_2S_2O_7$, and all other chemicals and solvents were purchased from Merck. Gelling water was HPLC grade and purchased from Spectrum.

Synthesis: Melem was synthesized following the reported procedure as in scheme S1a. All synthesis procedure of tricarboxylic acids were carried out according to scheme S1b. All the compounds were fully characterized by MALDI-TOF mass spectrometry, ^{1}H NMR spectroscopy (300 MHz), ^{13}C NMR spectroscopy (300 MHz), solid state NMR spectroscopy, FTIR and TGA.

Microscopic studies: The morphology of the gel was observed through an optical microscope (Leitz, Biomed) under perfectly crossed polarizer and taking the picture through a digital camera (Leica D-LUX 3). FESEM experiments were performed by placing a small portion of gel samples on a microscope cover glass. Samples were dried first in air and then in vacuum and coated with platinum prior to observation. After that micrographs were recorded by using a Jeol Scanning Microscope JSM-6700F.

Fluorescence microscopic and spectroscopic studies:Gel-phase material was placed on a glass microscope slide, dried, and examined under a fluorescence microscope (OLIMPUS BX-61) in 40X magnification. Fluorescence spectral studies of all the gel samples prepared in a sealed cuvette, were carried out in a Horiba Jobin Yvon Fluoromax 3 instrument. The gel samples were directly prepared in a quartz cell of 1 cm path length. Fluorescence excitation acquisition and emission acquisition were taken in slit width of 2/2 nm and scan rate was 0.2 s.

XRD studies: The WAXS studies of the dried gels were performed by a Seifert X-ray diffractometer (C- 3000) using nickel filtered Cu-Kα radiation with a parallel beam optics attachment. The instrument was operated at a 35 kV voltage and a 30 mA current and was calibrated with standard silicon sample. The samples were scanned from 2θ = 2° at the step scan mode (step size 0.03°, preset time 2 s), and the diffraction pattern was recorded using a scintillation counter detector.

Rheology studies: Rheological experiments were performed with an AR 2000 advanced rheometer (TA Instruments) using cone plate geometry in a Peltier plate. The plate diameter was 40 mm, with a cone angle of 2 degrees. Two types of experiments were performed: (i) by frequency sweep and (ii) by strain sweep methods. The frequency sweep experiments were made at 25°C at constant 1% strain. The strain sweep experiment was performed at 25°C at a constant frequency of 2 rads^{-1}.

FTIR studies: FT-IR spectra of MM, MEM, TPCA, BTA, CHTA and dried gels were recorded using KBr pellets of samples in an FTIR-8400S instrument (Shimadzu). All samples were diluted with KBr and pelletized prior to the experiment. Two independent experiments were performed for each sample.

2. Synthesis and characterization

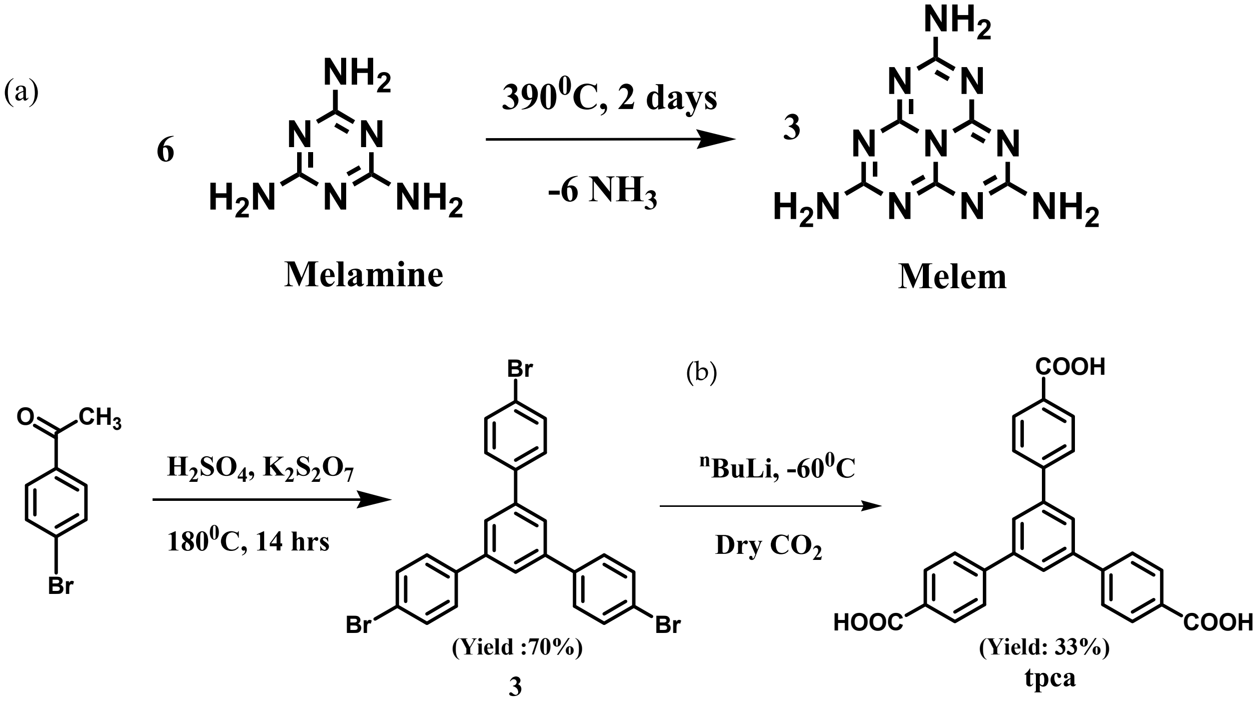

Scheme S1. synthetic procedure for (**a**) MEM and (**b**) TPCA

Synthesis of melem (MEM):[S1, S2] Melamine was initially weighted in porcelain and it was put into an oven at 390^{0}C for 2 days. Product was collected from the porcelain carefully. Yield: 35 %. Mass (MALDI-ToF): calcd 218.12 found 219.20 $(M+H)^+$

Synthesis of 1,3,5-Tri(4-bromophenyl)benzene (3): 4-Bromoacetophenone (10 g, 50.25 mmol), 0.5 mL of H_2SO_4(c) and $K_2S_2O_7$ (15 g, 59 mmol) were heated at 180°C for 14 hrs under nitrogen atmosphere. The resulting crude solid was cooled to room temperature and refluxed in 50 mL of dry EtOH for 1h and then cooled to room temperature.The solution was filtered and the resulting solid was refluxed in 50 mL of H_2O, gives a pale-yellow solid that was filtered. The crude product was dried under vacuum and recrystallized in $CHCl_3$. Yield :70%. ^{1}H-NMR (300 MHz, $CDCl_3$): δ = 7.53 (d, 6H), 7.60 (d, 6H), 7.68 (s, 3H) ppm. ^{13}C-NMR (300 MHz, $CDCl_3$): δ = 122.2, 125.1, 129,132.2, 139.7, 141.6 ppm.

Synthesis of 1,3,5-Tris(4-carboxyphenyl)benzene (TPCA): 1,3,5-tri(4-bromophenyl)-benzene (3 g, 5.52 mmol) was dissolved in 40 mL of anhydrous THF under N_2 atmosphere. The stirred solution was cooled to -60°C and a 1.6 M solution of n-BuLi in hexanes (10.5 mL, 16.3 mmol) was added dropwise. A red to light-green precipitation of the aryl lithium derivative was formed. Predried gaseous carbon dioxide was passed into the mixture at -60°C to give a colorless precipitate of the lithium salt. The mixture was allowed to warm and was quenched with 50% aqueous acetic acid. The solid product was filtered and recrystallized from acetic acid to give 0.8 g (33%) of white microcrystals. ^{1}H-NMR (DMSO-d_6): δ = 8.06 (s, 12 H), 8.09 (s, 3 H) ppm, ^{13}C-NMR (300 MHz, DMSO-d_6): δ = 125.6, 127.4, 129.9, 130.0, 140.8, 143.8, 167.2 ppm. Mass (MALDI-TOF) calcd 438.1 found 439.2 $[M+H]^+$. FTIR: 3071, 2985, 1691, 1608, 1419, 1318, 1294, 1245 cm^{-1}.

3. Solid state MAS NMR and MALDI-TOF of Melem

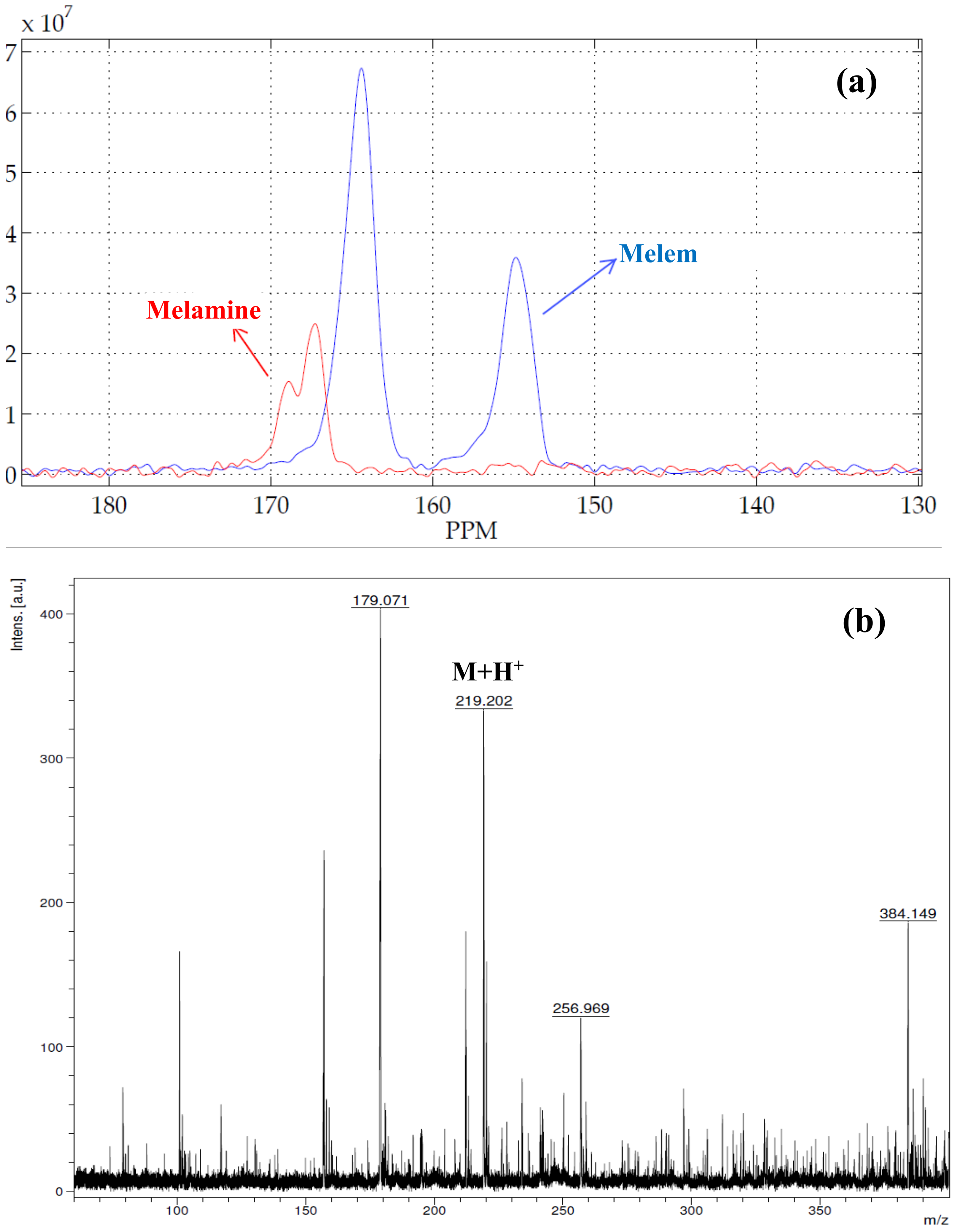

Figure S1. (**a**) Solid state MAS NMR of MM and MEM, and (**b**) Maldi-Tof mass spectra of MEM.

4. FTIR and TGA of Melem:

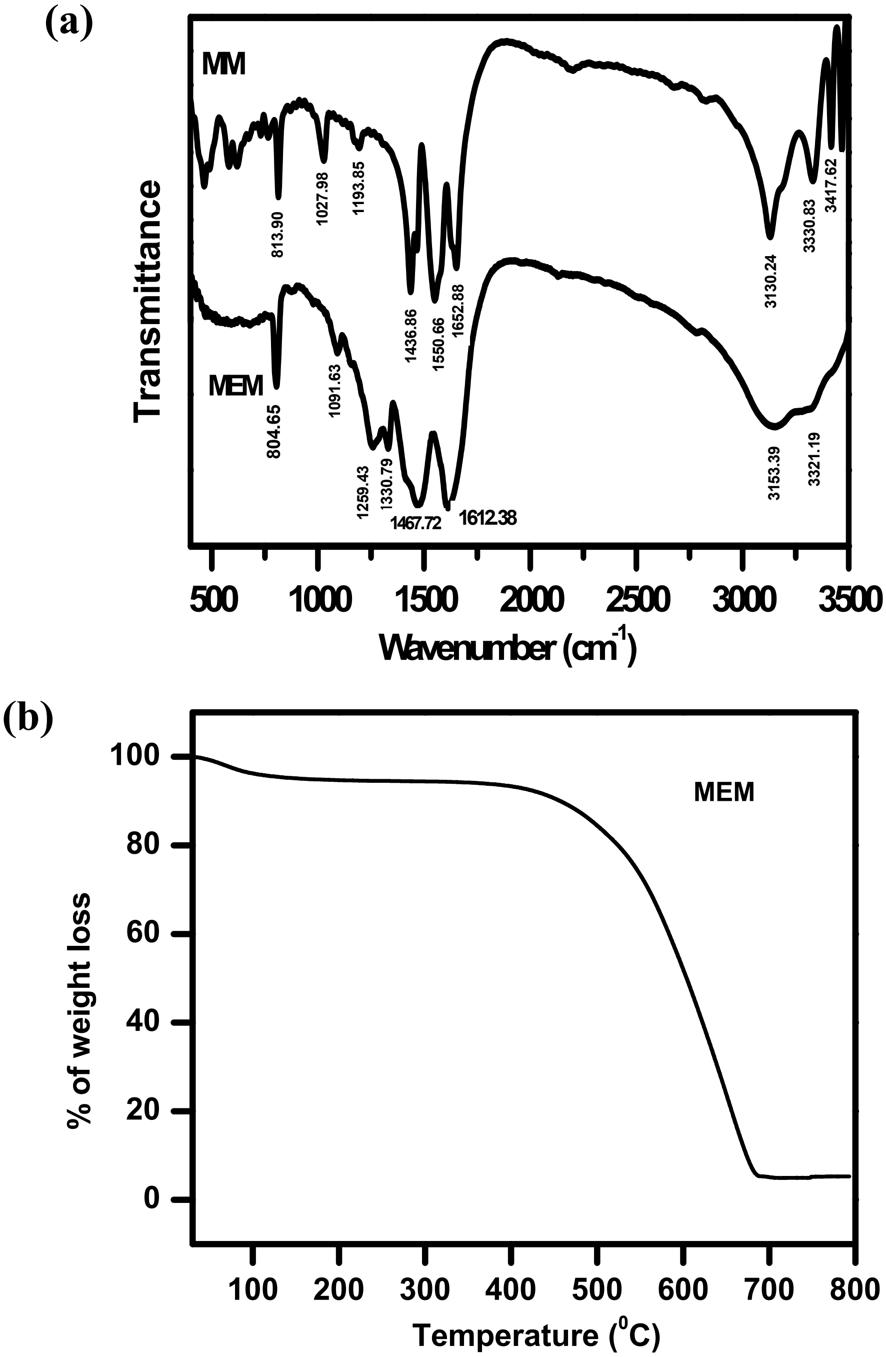

Figure S2. (**a**) FTIR spectra of MM and MEM, and (**b**) TGA characterization of MEM.

5. Fluorescence Microscopic images of different dried gel systems:

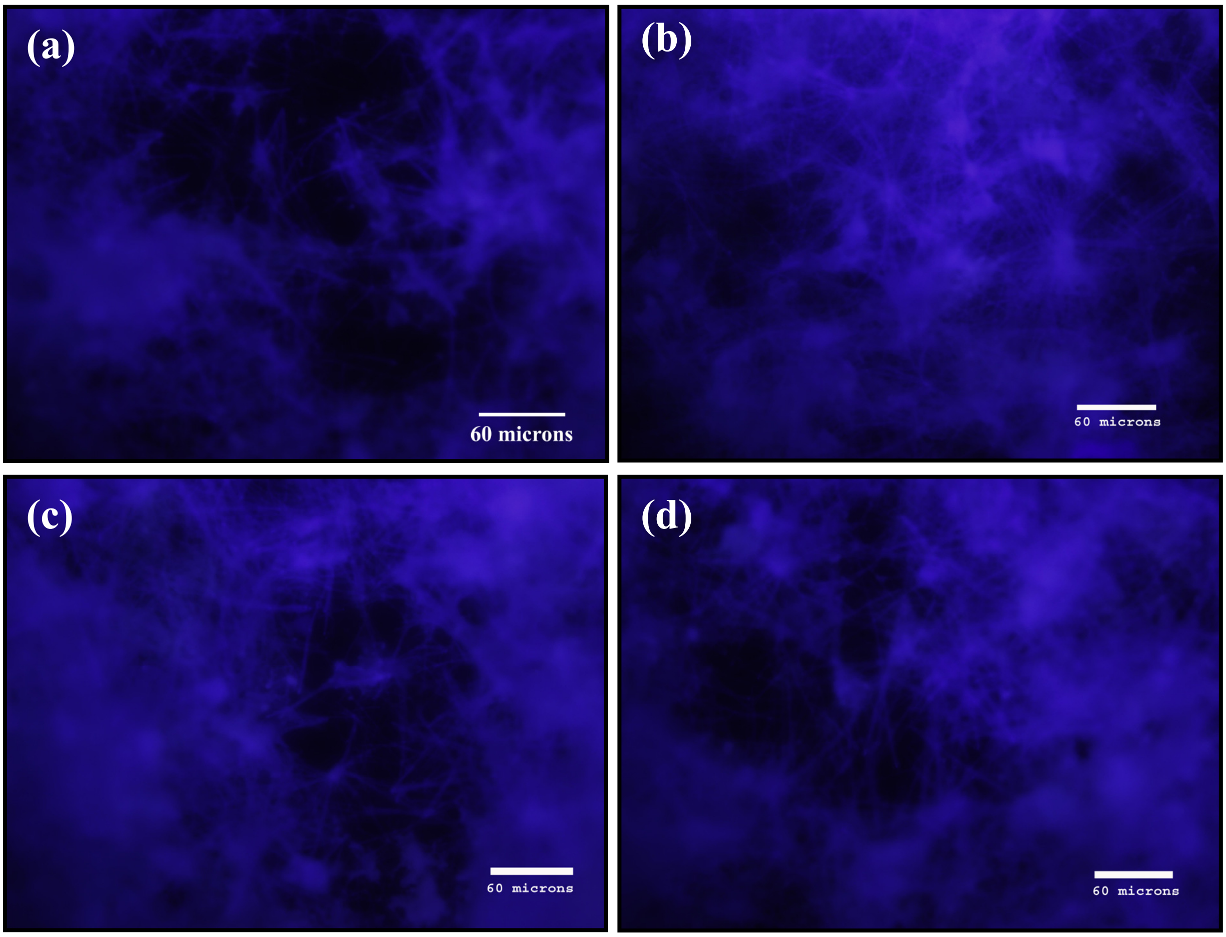

Figure S3. Fluorescence microscopic images of (**a**) MEM:TPCA 1:1, (**b**) MM:TPCA 1:1, (**c**) MM:BTA 1:1, and (**d**) MM:CHTA 1:1.

6. Observed Rheological values

Table S1. Estimation of elasticity and stiffness of hydrogel networks.

Samples	[Amine] : [Acid]	G′ (Pa) ± 100	G″ (Pa)± 40	Elasticity (G′-G″) ± 100	Stiffness (G′/G″) ± 1
[MEM]: [TPCA]	1:1	119,700	9884	109,816	12.11
	1:3	23,940	2471	21,469	9.68
	3:1	17,100	1098.22	16,001.78	15.57
[MM]: [TPCA]	1:1	9975	706	9269	14.12
	1:3	13965	847.2	13,117.8	16.48
	3:1	7125	441.25	6683.75	16.14
[MM]: [BTA]	1:1	9799	4776	5023	2.05
	1:3	63,030	32,460	30,570	1.94
	3:1	41,420	16,440	24,980	2.51
[MM]: [CHTA]	1:1	8155	2350.8	5804.2	3.46
	1:3	9961	3320	6641	3.00
	3:1	6363.12	2766.67	3596.45	2.29

Values of G′ and G″ are taken at 1Hz from the Figure 7.

7. Hydrogen bonding interactions between triacids and triamines:

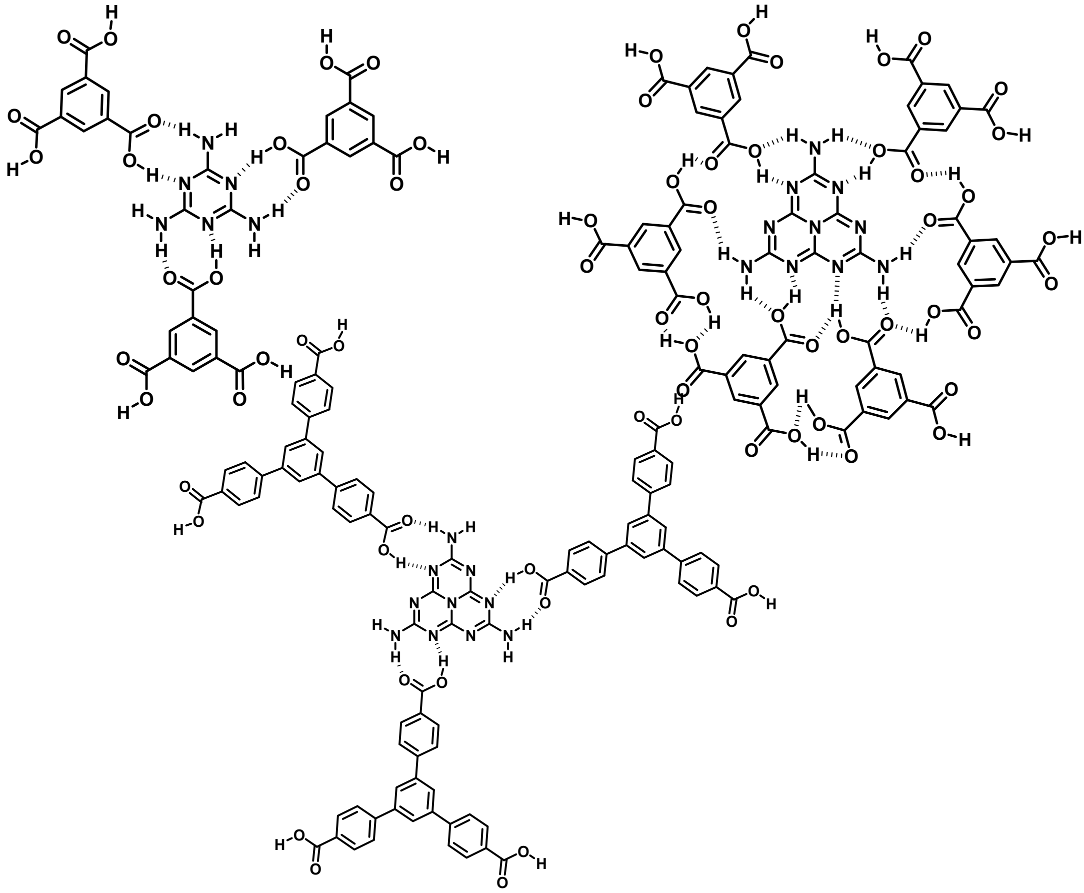

Figure S4. Hydrogen bonding interactions.

References

[S1] Holst, J. R.; Gillan, E. G. From Triazines to Heptazines: Deciphering the Local Structure of Amorphous Nitrogen-Rich Carbon Nitride Materials. *J. Am. Chem. Soc.* **2008**, *130*, 7373-7379.

[S2] Wirnhier, E.; Mesch, M. B.; Senker, J.; Schnick, W. Formation and Characterization of Melam, Melam Hydrate, and a Melam–Melem Adduct. *Chem. Eur. J.* **2013**, *19*, 2041–2049.
